# Research on the efficiency evaluation of the integration of technology and finance in China’s strategic emerging industries

**DOI:** 10.3389/fpsyg.2022.1103594

**Published:** 2023-01-18

**Authors:** Qing Yang, Kunqiang Zhao, Lanjuan Cao, Xiaohua Wang, Qizhe Guo, Xingxing Liu

**Affiliations:** ^1^School of Safety Science and Emergency Management, Wuhan University of Technology, Wuhan, China; ^2^School of Management, Wuhan University of Technology, Wuhan, China; ^3^Business School, NingboTech University, Ningbo, China

**Keywords:** technological innovation, financial support, three-stage DEA, multiple regression, development path

## Abstract

The combination of technology and finance has a great potential impact on regional social and economic development, and strategic emerging industries are the convergence of technological innovation and financial support, the development of which have great significance for industrial structure adjustment and industrial quality improvement. The geographical distribution of emerging strategic industries in China is uneven. We selected 18 provinces with relatively concentrated emerging industries. 206 STI board enterprises in 2020 were collected. A three-stage DEA model was used to measure the input–output efficiency of the integrated development of science and technology finance. Also, we used a regression model to examine the path of integrated development of science and technology innovation and financial support. The research finds that the development of strategic emerging industries has a large demand for financial support and obvious regional differences, and the government plays a strong guiding role in their development as well. At the end of the research, countermeasures and suggestions for the development of strategic emerging industries are given.

## 1. Introduction

The financial system provides the essential funding support for technology enterprises to facilitate the evolution of the industrial structure from lower to higher forms (Gravina and Lanzafame, 2020). The combination of technology and finance facilitates the upgrading of industrial structure. Studies on the impact of finance on industrial structure have been conducted long before, but the concept of “science and technology finance” has yet to be developed.

China’s technology finance emerged in the 1980s, it supports the development of technology enterprises in the form of technology loans and venture capital. In China’s financial system dominated by indirect financing, bank loans are still the main way for enterprises to obtain external financial support, but the characteristics of light-asset and high-risk enterprises make it difficult for high-tech enterprises to obtain bank credit support. The bank’s “credit grudging” tendency caused by the uncertainty of innovation activities makes China’s small and medium-sized high-tech enterprises face more serious “Macmillan defects” when conducting external financing (Kong and Zhou, 2020). In addition, in a survey of 885 institutional venture capitalists from 681 companies, Gompers et al. (2020) found that when selecting investments, VCs considered the management team more important than business-related characteristics such as product or technology, although there were meaningful cross-sectoral differences across company stages and industries. The risk management and dispersion function of the financial system can provide convenience for investors to avoid the high risks of technological innovation projects; at the same time, the high-yield nature of technological innovation and the profit seeking nature of the financial market are highly consistent, achieving the goal of capital value-added, which is conducive to the deepening development of finance; while the positive externality of technological innovation can reduce the information search cost of the financial system, thereby optimize the efficiency of capital allocation. The development of the financial system and the improvement of the financing environment can help to increase the innovation input and output of enterprises (George and Prabhu, 2003; Ayyagari et al., 2007), and the development of the financial market will significantly promote the efficiency of R&D investment (Chowdhury and Maung, 2012). Taking 32 developed and developing countries as empirical research object, Hus et al. (2014) found that the vigorous development of the stock market can effectively promote technological innovation, but the credit market may inhibit technological innovation.

The integration of technology and finance is essentially a financing behavior. Polzin et al. (2021) explored the investment and financing route in the area of energy transition technologies through a case study profiling decarbonized power system and found that both government and private investment require continuous investment. In order to obtain “technology premium,” the financial market will continue to pursue the most advanced technological achievements, provide financing services for cutting-edge technology research and development, and then promote technological progress. It can be seen that the combination of technology and finance has great potential to influence regional economic development. It can be seen that the integration of technology and finance has a significant impact on regional economic development. Some scholars have explored multiple dimensions of the impact relationship, for example, increasing R&D investment can effectively improve the production efficiency of enterprises (Lopez-Rodriguez and Martinez, 2014), new technological financial products such as bank technology loans have a positive effect on technological innovation (Ang, 2010), and technological finance has a significant positive impact on China’s international technological innovation, but there are regional differences in the promotion effect, showing a decreasing trend in the East, Middle and West (Wang and Guo, 2020). Other scholars have studied the impact of geographical location on technological innovation. Markus et al. (2022) pointed out that the development of regional innovation systems is influenced by the resources, actors, and institutional bases, and through an in-depth case study on the ferry electrification in western Norway, they found that geographic location and technological sustainability have a significant impact on technological change. Njos et al. (2020) further elaborated the interrelationship between geographic location and technological innovation, and found that neither the regional development status nor the technological characteristics can trigger the technological innovation level alone, but need to focus on the interaction between geography and technology in time and space. However, there is still a problem of financial repression in China’s financial system. Financial repression will exacerbate capital distortion and thus hinder technological innovation (Li and Ran, 2018). At the same time, if the project decision-making is wrong and technology loan is unbalanced, it will inhibit technological innovation (Sun and Song, 2020).

The integration of technology and finance has a far-reaching impact on industrial development. Rypestol et al. (2021) proposed a new theoretical framework to analyze the emergence and growth of industrial clusters. The framework focused on the role of assets in starting cluster development, and found that asset investment has an important impact on the adjustment of industrial structure. It also emphasized that regional development policies and regulations have an important impact on industrial clusters and structural optimization. Technological innovation has an important impact on the upgrading of industrial structure through such thresholds as technology market turnover, number of technical personnel, R&D expenditures and education expenditures (Wu, 2021). And this impact has regional heterogeneity (Liu et al., 2021; Li, 2022). Some scholars have also studied the role of national policies in promoting technology finance. Uyarra and Flanagan (2022) took the field of UAV technology as an entry point, and found that the formation of technological innovation and technical characteristics requires the support of national policies. What is more, the technological financial policy has a positive effect on the rationalization and advanced efficiency of the industrial structure (Hu and Liu, 2021), but it is still not prominent in the high-quality development (Feng and Qiu, 2021). Xu and Xu (2020) further pointed out the relationship between technology finance investment, technological innovation and economic growth, and believed that both technology finance and technological innovation are conducive to regional economic development, and there are lagging and spillover effects in the region.

The development of technology finance in China is based on the background of highly unbalanced regional development in China, and it is still at the exploratory stage. China has a vast territory, and there are significant differences in the development level of different regions. Generally, economically developed regions have a high level of high-tech development and sufficient reserves of technological talents. Industrial transformation, upgrading and transfer provide a certain opportunity for the coordinated development of social economy. Industrial transformation and upgrading are inseparable from technological innovation, and financial support is one of the necessary conditions for technological innovation. The integrated development of technology and finance can indirectly promote regional economic growth, and economic growth in turn will have a positive feedback effect on the development of technology and finance.

In the context of regional differences in the integration of technology and finance, analyzing the external manifestations and internal mechanisms of such regional differences and absorbing the development experiences of regions with high levels of integration will be conducive to expanding the high-level integration of technology and finance, which is a research gap in this field.

The integration of technology and finance is a possible way for transformation and upgrading of industrial structure and coordinated development of regional economy. Based on the analysis of technology and financial integration path of strategic emerging industries, this study collects data from 206 companies on the Science and Technology Innovation Board for strategic emerging industries in 18 provinces in China, uses the three-stage DEA model to measure the input–output efficiency of the integrated development of technology and finance, and uses the regression model to explore the path of integrated development of technological innovation and financial support, so as to promote the formulation and optimization of technological financial policies in various regions.

## 2. Construction of the framework model for the integration of technology and finance in strategic emerging industries

According to the Decision of the State Council on Accelerating the Cultivation and Development of Strategic Emerging Industries, strategic emerging industries, based on major technological breakthroughs and major development needs, playing a major leading role in the overall economic and social development and long-term development, are knowledge-and-technology intensive industries that consume less material resources, have great growth potential and have good comprehensive benefits. In the past 10 years, in addition to traditional bank loans and other financing methods, strategic emerging industries have made a lot of progress in terms of debt, equity financing model innovation and policy financial support, but there are still many constraints. Integration, agglomeration and ecologicalization are the new features in the development of strategic emerging industries during the “14th Five-Year Plan” period. In order to adapt to this new situation, it is necessary for the government to build a new industrial regulation system, change the industrial incentive measures characterized by “selectivity,” and further integrate all parties with finance as the hub.

As a high-tech industry, strategic emerging industry requires a large number of R&D investment, but a series of software and hardware environments restrict the improvement of the basic capabilities of independent innovation, making it difficult to better support the formation of an industrial development pattern led by independent innovation. Financial institutions generally hold an “ambiguous” attitude toward credit loans to strategic emerging industries (Wang et al., 2018). Specifically, on the one hand, they are constrained to make loans due to the regulations of national industrial policies, and on the other hand, the future business performance of strategic emerging industries is highly uncertain. With strong certainty, it is easy to generate bad debts and non-performing loan ratios, making financial institutions less motivated to lend, weakening the financial support, and restricting the transformation and upgrading of China’s industries and the establishment of innovation-driven economic growth mode.

### 2.1. Analysis on the development characteristics of strategic emerging industries

#### 2.1.1. High competition risk and uncertain returns

Affected by the accelerated reconstruction of the world’s political and economic structure, the trend of deglobalization will continue in the future, which will lead to the reconstruction of the global industrial cooperation pattern, the overall adjustment of the international division of labor system, and the intensification of international competition barriers in key links. China’s shortcomings in key core technologies and “stuck neck” links will become increasingly prominent, which will bring about serious hidden dangers to the security and stability of the industrial chain and supply chain of strategic emerging industries. Innovation is the internal driving force for the growth of strategic emerging industries, but technological innovation itself has uncertainties and high risks. These risks include not only the technical risks at the stage of technology research and development, but also the market risks at the stage of marketization when new products are not accepted by the market, as well as the institutional risks caused by factors such as changes in government policies and imperfect systems. Obviously, these high risks are contrary to the requirements of “safety, liquidity and profitability” pursued by commercial banks. In order to obtain financial support, enterprises often need to pay a risk premium that exceeds the necessary rate of return, which significantly increases the financing cost of enterprises. Although China has issued a large number of preferential policies to support the financing of strategic emerging industries, and commercial banks have also listed strategic emerging industry as a priority project category, in the actual credit process, however, products and projects that can effectively support strategic emerging industries are actually rare.

#### 2.1.2. Abundant intangible assets and weak financing guarantees

Banks and other financial institutions in China have set threshold conditions for the cash flow and fixed assets of enterprises in terms of credit business, requiring the credit subject to have a relatively stable cash flow, a clear profit model, a large number of fixed assets or sufficient collateral and guarantees. Strategic emerging industries are technology intensive industries, which are characterized by the low proportion of fixed assets in the balance sheet and the high proportion of intangible assets dominated by intellectual property rights. This makes many enterprises unable to meet the lending requirements of financial institutions due to the lack of effective mortgages and pledges, so they need to seek guarantee institutions from guarantees. At present, although there are many guarantee companies in China, their qualifications are uneven, their business varieties are single, their guarantee costs are high, and their guarantee loan term is short, which significantly increases the financing difficulty of strategic emerging enterprises. Leading enterprises lack the ability to integrate and lead the upstream and downstream enterprises, and have not formed a complete industrial chain, resulting in the lack of effective division of labor and coordination among enterprises and weak industrial organization. Small and medium-sized enterprises tend to be small in scale, whose products are concentrated in the middle and low-end and because of that there are relatively few products with high technology content, high added value and strong competitiveness. The intellectual property operation system of strategic emerging industry enterprises is not perfect, and the accumulation of core patents is insufficient.

#### 2.1.3. Multiple demand levels and mismatched entities

The main body of strategic emerging industries has various possibilities in terms of attributes, scale and development stage, and the capital needs and financing capabilities of different strategic emerging industries are also different, which requires a multi-level and diversified financial system to match. After more than 40 years of reform and opening up, China’s financial system has made great achievements, but it is still not perfect. Bank loans are still the most important channel for domestic social financing, the development of the capital market lags behind, the threshold of the stock market is high and the bond market is hard to underwrite, which has directly led to problems such as insufficient external financing, long-term capital shortage, and low financing efficiency in China’s manufacturing industry, especially strategic emerging enterprises. Consequently, the contradiction between single financing channel and diversified financing needs has become increasingly prominent.

Based on the above characteristics, compared with general enterprises, strategic emerging industries will face severe financing constraints and higher financing costs when financing, and financing constraints are one of the key issues that restrict the development of strategic emerging industries. The lack of financial support is the core problem that restricts the development of strategic emerging industries, which seriously restricts the development of strategic emerging industries in China.

### 2.2. Financial demand in three stages of development of strategic emerging industries

The development of emerging strategic industries can be divided into the stages of formation, growth and maturity, which correspond to the basic paths which includes the discoveries, technological innovations and industrialization of emerging industries (Xu and Yang, 2016).

#### 2.2.1. Analysis of capital demand in formative period

The formation period, also known as scientific discovery period. In this period, there are two main modes of industry formation mechanism. One is to imitate manufacturing and production by introducing foreign new process, new technology, new equipment, etc., to seize market opportunities. At the same time, the essence of foreign process and technology is constantly understood, familiar with and mastered through the production process, and on the basis of continuous summary and exploration, R&D capacity is effectively improved, so that new products, new processes and new technologies with independent intellectual property rights can be formed. The other mode mainly refers to domestic enterprises or scientific research units with abundant capital and R&D capabilities, using their own strength to conduct independent research and development of new products, new technologies and new processes, and fully grasp the core technology of a certain field. In the R&D process, make full use of technology penetration and diffusion to promote the development of strategic emerging industries. At this stage, the product R&D and technological innovation of strategic emerging industries have the characteristics of long R&D cycle and large investment in material, human and financial resources and at the same time have great uncertainty, which cannot guarantee the success rate of R&D. Therefore, there are large investment risks. However, enterprises have high risk appetite for capital, and the financing risk of enterprises is relatively high. What they need is capital that can be used for a long time.

On the whole, China’s strategic emerging industries, who have obvious deficiencies in development experience, self-capacity, public services and other aspects, are still “new recruits” in the international market and the capacity needs to be strengthened. The financial management ability in the initial stage is relatively weak. On the one hand, enterprises mainly rely on the existing bank account services, payment and settlement, international finance, maintenance and appreciation of domestic and foreign currency capital and other general-purpose products to carry out financial activities. They rarely put forward customized needs and more consider the convenience of existing banking products.

#### 2.2.2. Analysis of capital demand in growth period

With the development of strategic emerging industries, strategic emerging industries have entered the growth period. The enterprises have begun to take shape, the technical level of the industry has been improved, and the market risk has been reduced. The enterprises have made breakthrough R&D achievements in new technologies, new products and new processes, have fully mastered the core technologies in a certain field, and has a first-mover advantage, which can effectively seize the business opportunities in the market and effectively improve the economic efficiency. Enterprises at this stage mainly carry out technological innovation, expand market share, improve the market competitiveness of products and cultivate the core capabilities of enterprises, enabling themselves achieve healthy and rapid development. Meantime, at this stage, the profitability of enterprises has been greatly improved, the sources of funds and financing channels are relatively smooth, the financing modes that enterprises can adopt are more diverse, and the risk appetite for capital is reduced. This kind of industry can be favored by many investors and manufacturers, and will also gather a large number of financial, material and human resources to effectively promote the rapid growth of the scale of this industry. The main task of emerging industries at this stage is that enterprises need to expand production scale, expand market sales channels, improve product quality and improve management. Therefore, it is facing the financial pressure of rapid expansion, such as building new factories, purchasing equipment, introducing technology, training employees, etc., all of which require a lot of investment. At the same time, it also has strong financial attractiveness owing to its broad market prospects and ideal investment benefits.

#### 2.2.3. Analysis of capital demand in mature period

At this stage, on the one hand, the market capacity has tended to be saturated due to the development and expansion of the industrial scale; on the other hand, the social and economic benefits of enterprises have been effectively improved and tend to be stable, the social awareness of enterprises has also been gradually established. In the national economic system, such industries have become increasingly mature and the proportion is relatively stable. At this stage, the industry needs to intensify the research on technological innovation and product upgrading, effectively promote the merger and reorganization of enterprises, and realize the scale of the industry. Therefore, the capital demand at this stage mainly comes from two aspects: enterprise merger and reorganization and product technology upgrade, and has the characteristics of stable income and low investment risk. After entering the industrial maturity period, the enterprise has significantly improved its technical level, significantly reduced the market risk, continuously enhanced its profitability because of having a good financial situation and a relatively low risk (Zhu, 2021). The main task of enterprises is to innovate the technical level, organizational structure and management mode, and realize the transformation of strategic emerging industries. Enterprises need wider financing channels to meet higher financing needs. Credit established on the basis of stable cash flow and market share can enable enterprises to adopt lower cost financing methods, including issuing bonds, issuing stocks at a high price, more favorable loan terms and higher loan amounts from commercial banks.

**Figure 1:**
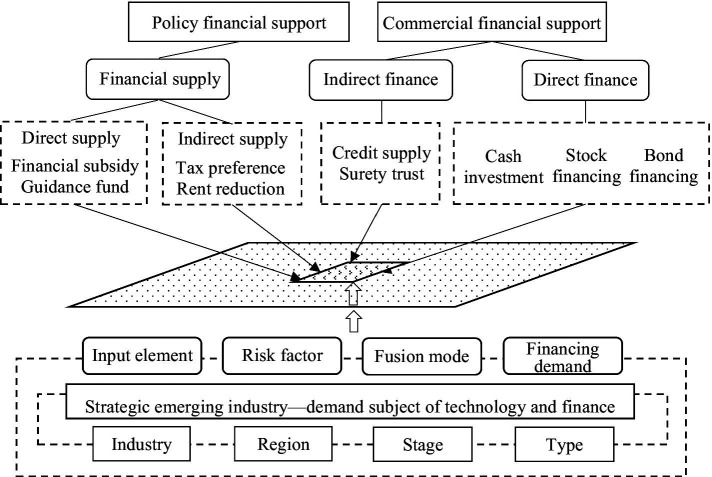
Framework model for the integration of technology and finance in strategic emerging industries.

To sum up, according to the development characteristics of the above-mentioned strategic emerging industries and the analysis of financial needs at each development stage, a framework model for the integration of technology and finance can be constructed, as shown in Figure 1.

## 3. Construction of efficiency evaluation model for the integration of technology and finance of strategic emerging industries

Shahzad et al. (2021) argued that the relationship between debt financing and corporate innovation is the most controversial debate in modern corporate finance research, and there is a need to explore the role and impact of the effective use of debt on R&D intensity and technology investment. They used the number of patents to measure corporate innovation and conducted an empirical study using regression analysis, and the empirical results verified that the link between debt financing and corporate innovation is inverted U-shaped, which confirms the Kuznets curve hypothesis. However, the study does not resolve the cause of regional differences between finance and technology, i.e., why the regional finance and technology associations are at different levels of the inverted U-shape. Rajeswari and Krishnan (2021) dissected theoretical and case studies on fintech drivers, the shortcomings of traditional financial services, and the role of technological advances, and also discussed the main issues hindering fintech investments, such as technological innovation prospects, investment management, customer management, and regulation. However, the study only analyzes the prospects of the integration of technology and finance in terms of theory and current situation, and the quantitative research is slightly inadequate.

Due to the prominent contradiction between the “effectiveness, precision and continuity” of the deep integration of technology and finance and the characteristics of technology finance, the policy promotion of technology finance has great ambiguity. At the same time, the integration point of technology finance is difficult to locate and the feedback cycle of technology finance is unstable. In order to quantitatively analyze the current situation of the two-way dynamic matching of supply and demand between financial supply subjects and financial demand subjects, it is necessary to consider the subjective and objective transformation conditions such as environment and management in efficiency measurement, and conduct multiple rounds of evaluation to ensure that the two-way dynamic matching of supply and demand in the deep integration of technology and finance is stable and effective.

There are two effective tools for evaluating relative efficiency: Stochastic Frontier Analysis (SFA) of parametric method and Data Envelopment Analysis (DEA) of nonparametric method (Cullinane and Song, 2006). Among them, SFA is limited to the efficiency evaluation of a single output, while DEA is based on the concept of “relative efficiency” and evaluates the relative effectiveness or benefit of the same type of units (departments) according to multi-indicator input and multi-indicator output (Charnes et al., 1978). There are more than one output items to measure the two-way dynamic matching model of supply and demand of technology finance (Yu, 2015; Du et al., 2016), which determines that the index system measured by the two-way dynamic matching model of supply and demand of technology finance is multi-input-multi-output type.

DEA method is superior to other multi-objective evaluation methods in dealing with the problem of multiple inputs and multiple outputs. When evaluating the decision-making unit, the optimal weight is obtained according to its actual data, and there is no need to make any assumptions about the weight, so that it has strong objectivity.

Peng and Zheng (2021) used stochastic frontier analysis to measure the energy efficiency of Chinese provinces from 2001 to 2017, and tested and analyzed the promotion effect of green finance on energy efficiency and the mediating effect of green technological innovation. The results show that green finance can significantly improve energy efficiency. The impact of green finance on energy efficiency is greater in provinces with rich resource endowments, high levels of economic development, and high degrees of marketization. Peng and Zheng (2021) made assumptions and conducted Robustness Tests and Heterogeneity Analysis for the measured results and obtained plausible conclusions. In contrast, our study focuses on exploring the differences in technology and financial integration between regions.

Our study adopts a Three-stage DEA model, which overcomes the defects of the traditional DEA model, and eliminates the impact of environmental variables, random interference, management inefficiency and other factors on the deep integration of technology and finance, so as to more accurately and reliably measure the input–output efficiency.

In the first stage, we adopt an input-oriented BCC model with variable returns to calculate the technical efficiency (TE), pure technical efficiency (PTE), and scale efficiency (SE) of each decision-making unit (Yang et al., 2013; Zhao and Guan, 2015). Referring to the research of Banker et al. (2017), the model is expressed as:


(1) $$\min\left[\theta -\varepsilon \left(e^{T}s^{-}+e^{T}s^{+}\right)\right]$$

(2) $$s.t\{\begin{array}{c} \overset{X}{\underset{i=1}{\sum }}\lambda_{i}Y_{i}+S^{-}=\theta X_{ik}\\ \overset{X}{\underset{i=1}{\sum }}\lambda_{i}Y_{i}-S^{+}=Y_{ik}\\ \lambda_{i}\geq 0,S^{+}\geq 0,S^{-}\geq 0\\ \overset{X}{\underset{i=1}{\sum }}\lambda_{i}=1,i=1,\ldots ,N \end{array}$$

Where X refers to the input indicator variable matrix of each city, Y refers to the corresponding output indicator variable matrix, K represents the number of decision-making units, $\lambda_{i}$ refers to the weight of the *i*-th input variable, $S^{-}$ and $S^{+}$ are input slack variables and output slack variables respectively, ε is an arbitrary infinitesimal positive number, and θ is the input–output efficiency value of the two-way dynamic matching model of supply and demand of technology finance.

The DEA-BCC model can scientifically measure the efficiency value (technical efficiency) and its related decomposition values (pure technical efficiency, scale efficiency), its calculation formula is as follows:


$$\begin{array}{c} \mathrm{Technical}\;\mathrm{Efficiency}\left(\mathrm{TE}\right)=\mathrm{Pure}\;\mathrm{Technical}\;\mathrm{Efficiency}\left(\mathrm{PTE}\right)\\ \times \mathrm{Scale}\;\mathrm{Efficiency}\left(\mathrm{SE}\right) \end{array}$$

Pure Technical Efficiency (PTE) reflects the production efficiency of input factors of DMU at a certain optimal scale. Scale efficiency (SE) refers to the effect of industrial structure on output units through optimal allocation. Technical efficiency (TE) refers to the ratio of the actual output of the decision-making unit to the maximum output for a given set of input elements, reflecting the ability of the decision-making unit to obtain the maximum output under the given input. If TE is equal to 1, the enterprise is located on the production frontier (the production frontier refers to the set of efficient input–output vectors of the decision-making unit under a certain technical level, that is, the set of the maximum output under a given input or the minimum input under a given output), whose PTE and SE are both 1.

In the second stage, we use the SFA method to filter out the influence of environmental factors and inefficient management, so that all decision-making units are in the same external environment, and then take the input redundancy obtained in the first stage analysis as the explanatory variable, and the environmental variable and the mixed error term as the explained variable. The SFA model estimates the technical efficiency of the decision-making unit by decomposing the error term. The error term is divided into two parts, one representing random error and the other representing technical inefficiency. The following SFA regression function is constructed:


(3) $$S_{ni}=f\left(Z_{i};\beta_{n}\right)+v_{ni}+\mu_{ni},i=1,2,\ldots ,I;n=1,2,\ldots N$$

Where $S_{ni}$ is the redundancy of the *n*-th input variable of the *i*-th decision-making unit, $Z_{i}$ is the environmental variable, $\beta_{n}$ is the coefficient of the environmental variable, and $v_{ni}+\mu_{ui}$ is the mixed error term, where ν ~ N (0， $\sigma_{\nu }^{2}$ ), μ ~ N (0， $\sigma_{\mu }^{2}$ ).

In the third stage, we also adopt the traditional DEA-BCC model, input the adjusted input data into DEAP 2.1, and calculate the technical efficiency, pure technical efficiency and scale efficiency of each decision-making unit again. At this time, the efficiency has eliminated the influence of environmental factors and random factors, which is relatively true and accurate.

The study uses multiple regression model to analyze the development of financial support for strategic emerging industries, and tests the reasons for this difference through empirical research, so as to find an optimization path.

Assuming that economic growth under technological innovation conforms to the C-D production function (Wang and Sun, 2020), the production frontier function is Y=(X,β) , where X is production-related factor, such as manpower, capital, technology, etc., which can be converted into


(4) InY=α+∑βlnX+u

The regression model is constructed as follows:


(5) $$InU=\alpha +\beta_{1}\ln R+\beta_{2}\ln S+\beta_{3}\ln G+\beta_{4}\ln P+\mu$$

Considering the efficient operation of the financial system, the explained variable of the regression model is lnU , and U is the unit efficiency output, which is set as $\frac{I}{E}$ . I is the operating income of strategic emerging enterprises; E is the comprehensive efficiency value (technical efficiency TE obtained from the DEA operation result); R is the proportion of the R&D personnel of strategic emerging enterprises, which is used to measure the human resource investment of the strategic emerging enterprise; S is the ratio of R&D investment to operating income, which is used to measure the capital investment of strategic emerging enterprises; G is government subsidies, which is used to measure government financial investment; and P is the amount of patent authorization, which is used to measure the technological strength of strategic emerging enterprises.

## 4. Selection of indicators and data sources

### 4.1. Selection of input and output indicators and environmental variables

By analyzing the literature on the construction of an indicator system for the efficiency of combining finance and technology, it is found that the existing literature mostly uses the total amount of indirect financing, the amount of government subsidies, R&D investment, R&D personnel, the number of patents granted, net profit, the amount of technology market contract turnover, and the proportion of new product sales revenue to product sales revenue as indicators to measure the output–input ratio (Li and Cheng, 2013; Pan and Zhang, 2018).

Since there is a lack of data on angel investment, venture capital and bank loans specifically for strategic emerging enterprises, this paper focuses on drawing on the development of listed strategic emerging enterprises and selects the number of R&D personnel, the ratio of R&D investment to operating revenue and government subsidies as input indicators, and patents and operating revenue as output indicators. Among them, the number of R&D personnel also represents to a certain extent the investment in technology innovation of the enterprises (Song and Liu, 2021), so the ratio of R&D investment to operating revenue, the amount of government subsidies and the number of R&D personnel are selected as the technology financial input, the number of patents granted as the innovation output of strategic emerging enterprises, and the operating revenue as the operating output of strategic emerging enterprises.

The selection of environmental variables is a key step in the three-stage DEA method. In order to satisfy the “separation hypothesis,” environmental variables need to be selected as factors that have an impact on the operational efficiency of enterprises but cannot be subjectively controlled. Combining the characteristics of listed enterprises in strategic emerging industries and the research results of many scholars, this paper summarizes the external factors affecting the operational efficiency of enterprises as the level of regional economic development and the current situation of the regional population.

The level of regional economic development. Strategic emerging enterprises are generally a microcosm of a region’s economy, which reflects the direction and focus of the region’s industrial layout. As representatives of the transformation and high-quality development of the national economy, strategic emerging enterprises must consider the impact of the level of regional economic development on them when examining the issue of their technical efficiency. Relevant studies have found that there is a positive relationship between regional GNP and enterprise-scale expansion (Ji and Kong, 2014). In this paper, regional GDP is selected to measure the impact of the level of regional economic development on the technical efficiency of strategic emerging enterprises.

The current state of the regional population. People are both producers and consumers, and the impact of population on the development of strategic new enterprises is mainly reflected in these two aspects. The population that is the producer is the working population, and in order to act as a working population, this population must have a certain level of physical strength, skills and experience combined to form a labor force. Although the population with this capacity to work is only a part of the regional population, its production serves the population of the region as a whole. Therefore, this paper examines the impact of the population size of each region on strategic emerging enterprises, as shown in Table 1.

**Table 1:** Input–output indicators for the integration of technology and finance in strategic emerging enterprises.

Measurement category	Measurement indicators
Inputs	R&D investment as a percentage of operating revenue (%)
	Amount of government grants (RMB million)
	Number of R&D staff as a percentage (%)
Outputs	Number of patents granted (pieces)
	Operating income (RMB million)
Environment variables	GDP (RMB billion)
	Number of the resident population (10,000)

### 4.2. Data sources

The Science and Technology Innovation Board (STIB) is the first over-the-counter market in China to implement a registration system. It mainly serves technology innovation enterprises that are in line with national strategies, breakthroughs in key core technologies and high market recognition, and focuses on supporting high-tech industries and strategic emerging industries such as new generation information technology, high-end equipment, new materials, new energy, energy conservation and environmental protection as well as biomedicine. Therefore, enterprises listed on the Science and Technology Venture Exchange are considered strategic emerging enterprises in this study.

CSMAR was used to find the list of enterprises listed on the STB in the 18 provinces and cities with a more dense sample of strategic emerging industries in 2020, to determine the list of enterprises in each province and city based on the criteria for identifying technology-based enterprises, strategic emerging industries and technology-based SMEs, and to eliminate enterprises lacking the number of R&D personnel and the amount of government subsidies, and finally screen out 206 strategic emerging enterprises on the STB (Table 2).

**Table 2:** Correlation analysis.

		lnU	lnR	lnS	lnG	lnP
lnU	Pearson correlation	1	0.006	0.013	0.766^**^	−0.035
	Significance (two-sided)		0.983	0.960	0.000	0.890
	N	18	18	18	18	18
lnR	Pearson correlation	0.006	1	0.462	0.017	−0.058
	Significance (two-sided)	0.983		0.053	0.945	0.819
	N	18	18	18	18	18
lnS	Pearson correlation	0.013	0.462	1	0.027	0.108
	Significance (two-sided)	0.960	0.053		0.915	0.671
	N	18	18	18	18	18
lnG	Pearson correlation	0.766^**^	0.017	0.027	1	0.052
	Significance (two-sided)	0.000	0.945	0.915		0.837
	N	18	18	18	18	18
lnP	Pearson correlation	−0.035	−0.058	0.108	0.052	1
	Significance (two-sided)	00.890	0.819	0.671	0.837	
	N	18	18	18	18	18

^**^Significantly correlated at the 0.01 level (two-sided). The correlation analysis reveals that U and G are significantly correlated.

## 5. Empirical analysis

### 5.1. DEA three-stage analysis

Using the three-stage input-oriented DEA model, the technical efficiency (TE), pure technical efficiency (PTE) and scale efficiency (SE) of 206 strategic emerging enterprises in 18 provinces and cities were measured in the three stages of development with the help of DEA 2.1 software.

Phase I: Evaluation results of the efficiency of technology-finance integration in strategic emerging industries based on traditional DEA models.

**Figure 2:**
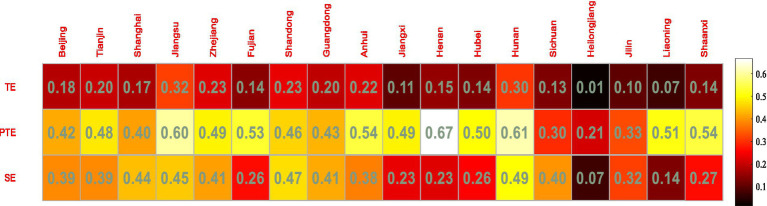
Strategic emerging industries technology and finance stage 1 DEA Evaluation Value.

The mean values of TE, PTE, and SE were used to reflect the efficiency of technology and finance integration in strategic emerging industries in each province and city. The results of the empirical analysis are shown in Figure 2.

The mean values of TE, PTE and SE in the 18 provinces and cities are 0.212, 0.479, and 0.404, respectively. From Figure 2, it can be seen that on the whole, the PTE and SE in the 18 provinces and cities are relatively low, with less than 50% of the regions above the mean value. The efficiency of financial support for the development of strategic emerging industries needs to be improved, and is far from the production frontier, so the direction of efficiency improvement should be balanced to raise PTE and SE and Improve the technology and management level of enterprises.

Specifically, among the 18 provinces and cities, Jiangsu and Hunan had the highest TE values and Heilongjiang and Jilin had the lowest TE values, with the overall TE values ranging from 0.072 to 0.316, a wide range. As for the PTE values, Heilongjiang had the lowest PTE value of 0.213, while the rest of the provinces and cities had PTE values in the range of 0.296 to 0.666. And among the SE values, Heilongjiang had the lowest SE value of 0.068 and Hunan had the highest SE value of 0.494, while the rest of the regions had SE values ranging from 0.136 to 0.472.

The technical efficiency of financial support for the development of strategic emerging industries is the highest in Jiangsu and Hunan, with 0.316 and 0.303 respectively, and the best financial support for the development of strategic emerging industries. Both Jiangsu and Hunan have high PTE and SE. Suzhou, as one of the first pilot regions in China to integrate technology with finance, has gradually developed a diversified model of integrating technology with finance, taking into account the characteristics of regional economic development. As the economic powerhouse of Jiangsu province, Suzhou has driven the development of strategic emerging industries in other cities in the province. Hunan, on the other hand, has in recent years successively implemented various types of forced responsibility promotion programmes to promote and stimulate the development of the province’s strategic emerging industries.

The technical efficiency of financial support for the development of strategic emerging industries in Anhui and Hubei is at a medium level within the 18 provinces, with an overall range between 0.103 and 0.229. The main reason is that most of the strategic emerging industries are information technology, high intelligence equipment and other industries, while most provinces and cities in such areas still have certain problems in financing and technological innovation in such industries, and there is an urgent need to combine the province’s technology. There is an urgent need to implement technology innovation policies with local characteristics, taking into account the characteristics of the province and city’s endowment of technology and financial resources.

The technical efficiency of financial support for the development of strategic emerging industries is the lowest in Heilongjiang and Liaoning, at 0.015 and 0.072, respectively. As can be seen from Table 3, both the PTE and SE values in Heilongjiang are low, i.e., both the technical efficiency and scale efficiency of financial support for strategic emerging industries in the province are low, while the SE value in Liaoning is low, i.e., the scale efficiency of financial support for strategic emerging industries in the province is low, mainly due to the fact that Liaoning and Heilongjiang’s lower level of economic and technological development, the smaller number of strategic emerging enterprises, their lower financing capacity, and the low transformation capacity of the enterprises’ technological efficiency.

**Table 3:** Regression coefficients and test results of the development path of strategic emerging industries.

	Non-standardized coefficients		Standard factor	*t*	Sig.	Covariance statistics	
	*B*	Standard deviation	Trial version			Tolerance	VIF
(Constant)	8.775	1.612		5.443	0.000		
lnR	−0.023	0.290	−0.016	−0.078	0.939	0.774	1.291
lnS	0.003	0.083	0.007	0.037	0.971	0.768	1.302
lnG	0.636	0.146	0.770	4.344	0.001	0.997	1.003
lnP	−0.067	0.157	−0.077	−0.429	0.675	0.971	1.030

**Figure 3:**
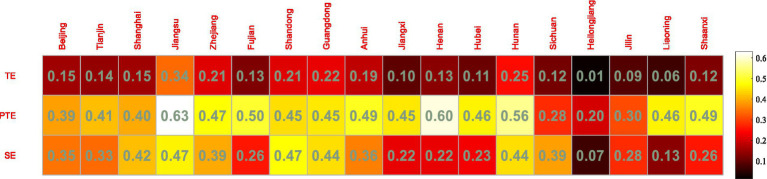
Three-stage DEA evaluation of technology and finance in strategic emerging industries.

The SFA method was used in the second stage to optimize the explanatory and explained variables of the DEA, and the third stage carried out the input variable adjusted DEA evaluation to obtain Figure 3.

**Figure 4:**
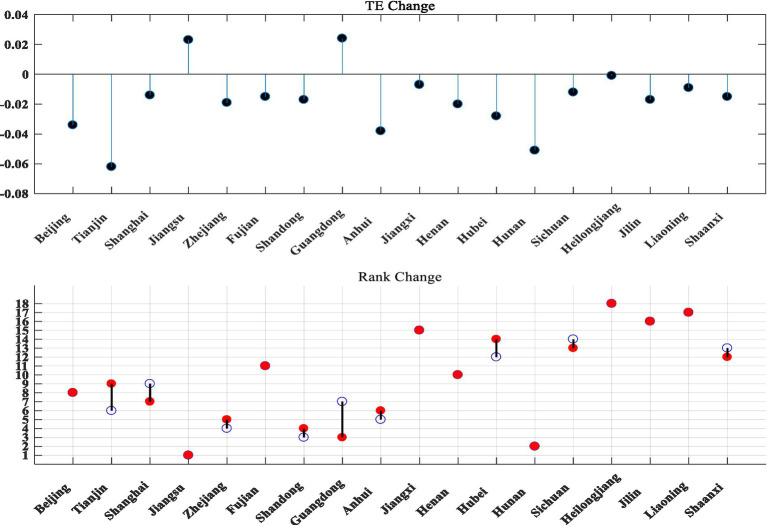
Three stages of TE change in technology and finance in strategic emerging industries. The blue hollow circles in the chart are the first stage rankings, the red solid circles are the third stage rankings.

**Figure 5:**
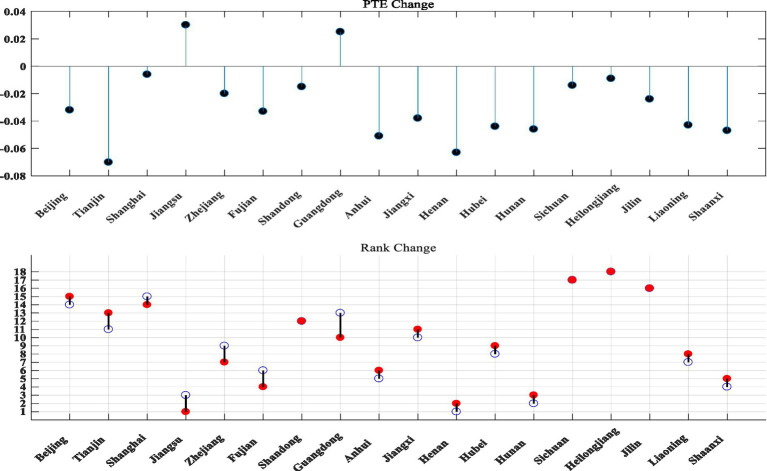
Changes in the three stages of PTE for technology and finance in strategic emerging industries.

**Figure 6:**
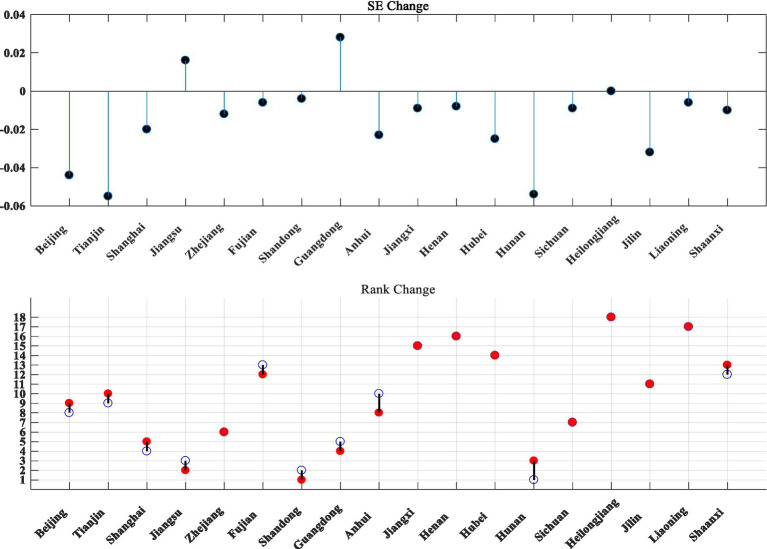
Three stages of SE change in technology and finance in strategic emerging industries.

As can be seen from Figure 3, after excluding the influence of environmental variables and random factors, the comprehensive technical efficiency value, pure technical efficiency value and scale efficiency value of strategic emerging industries in 18 provinces and cities have risen and fallen. The ranking of Guangdong Province changed significantly, with the comprehensive technical efficiency dropping eight places and the scale efficiency dropping five places. After adjusting the data of the original inputs, the rankings of all provinces and cities have risen and fallen to different degrees (see Figures 4–6 for details).

The mean TE value is 0.204, which is somewhat lower; among the 18 provinces and cities, Jiangsu has the highest TE value of 0.339, while Heilongjiang and Liaoning still have the lowest TE values at 0.014 and 0.063 respectively, and the rest of the provinces and cities have TE values ranging from 0.107 to 0.253.

The mean PTE value was 0.471, with no significant variation. Jiangsu had the highest PTE value of 0.630, Heilongjiang had the lowest PTE value of 0.204, and the rest of the provinces and cities had PTE values ranging from 0.282 to 0.604.

The mean SE value is 0.395, which also decreases to a certain extent. This indicates that the low overall efficiency of technological innovation in strategic emerging industries is mainly due to the inadequacy of both technological innovation efficiency and the scale of technological innovation activities. Heilongjiang has the lowest SE value of 0.068, Shandong has the highest SE value of 0.468, and the rest of the regions have SE values ranging from 0.130 to 0.440.

Overall, Jiangsu still has the highest TE values after the adjustment and has increased compared to the first stage, mainly because both PTE and SE in Jiangsu have increased after the adjustment, while PTE and SE in more provinces and cities have decreased to a certain extent after the adjustment, therefore the combined TE values in Jiangsu have increased to a certain extent compared to the first stage. The TE value for Heilongjiang remained the lowest after the adjustment of input variables, with very little change compared to the first stage, mainly because the PTE and SE values for Heilongjiang remained the lowest after the adjustment, with no change compared to the first stage, and therefore the combined resulting TE value for Heilongjiang remained the lowest.

### 5.2. Comparative analysis of different regions

**Figure 7:**
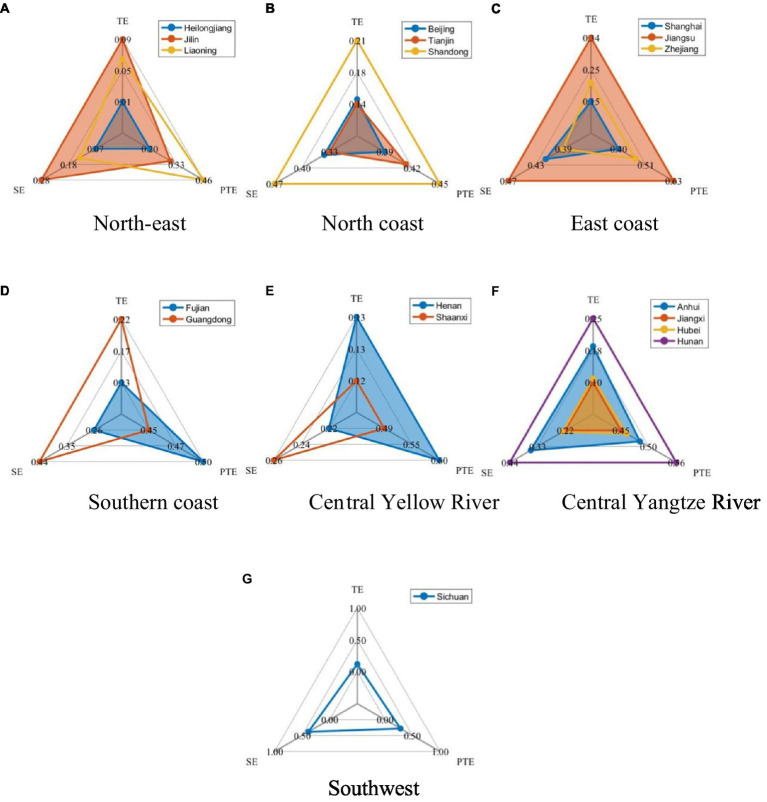
Three-stage DEA comparison by region. **(A)** North-east. **(B)** North coast. **(C)** East coast. **(D)** Southern coast. **(E)** Central Yellow River. **(F)** Central Yangtze River. **(G)** Southwest.

Referring to the 2016 State Council Development Research Centre’s “Strategies and Policies for Coordinated Regional Development” proposed method of dividing economic zones, the 18 provinces and cities can be divided into the northeast (Liaoning, Jilin, Heilongjiang), northern coast (Beijing, Tianjin, Shandong), eastern coast (Shanghai, Jiangsu, Zhejiang), southern coast (Fujian, Guangdong), middle reaches of the Yellow River (Henan, Shaanxi), middle reaches of the Yangtze River (Anhui, Jiangxi, Hunan, Hubei), and Southwest China (Sichuan), and then carry out the comparative analysis of the same region. The three-stage DEA comparisons for these regions are shown in Figure 7.

As can be seen from Figure 7, the development efficiency of strategic new industries in the three northeastern provinces varies widely. Among them, Jilin Province and Liaoning Province are higher, and both have different sources of advantage. Liaoning Province has higher pure technical efficiency, while Jilin Province relies on scale efficiency. Heilongjiang, on the other hand, needs to increase its support for strategic emerging industries and continuously optimize and upgrade them.

Among the northern coastal regions, Shandong’s strategic new industry development efficiency is at a high level, compared to Beijing and Tianjin’s moderate strategic new industry development, reflecting the political and economic integration of the two regions.

Among the eastern coastal regions, Shanghai, Zhejiang and Jiangsu are all at a high level. As the first province in China to deploy the development of strategic emerging industries, Jiangsu Province put forward the “Emerging Industry Doubling Plan” as early as August 2010, and identified six emerging industries for key development, which has a first-mover advantage.

In the southern coastal region, Guangdong’s strategic new industries are at a higher level of development and are at a clear advantage in terms of scale and efficiency, which is closely linked to the high-tech industries led by the Shenzhen.

In the central Yellow River region, Henan has a more significant impact on the development of strategic new industries in terms of pure technical efficiency, while Shaanxi is more of a certain scale, but large rather than strong.

In the central Yangtze River region, the development of each province is more coordinated, with Hunan and Anhui having better development of strategic new industries, but there are few differences between provinces.

The southwest region, with only Sichuan province in the sample, has a more balanced pure technical efficiency and scale efficiency in the development of its strategic new industries, but the overall efficiency is not high and needs further policy promotion. The remoteness of the southwest and northwest regions, the complex topography, the highland climatic conditions and the ecological environment of the mountain grasslands make these places less populous and less economically active.

In general, the eastern and southern coasts are in a better position to develop strategic emerging industries, the north is in a steady state, and the central Yangtze River region is developing in a balanced manner and is expected to become a gathering area for strategic emerging industries. There are certain opportunities in the central and southwestern Yellow River.

### 5.3. Path creation simulation

The average value of 206 strategic emerging enterprises in 18 provinces and cities was used for calculation. To initially understand whether there is correlation between the independent and dependent variables, and whether there is multicollinearity between the independent variables, Pearson correlation analysis was first conducted for each variable.

Based on Table 4, an F-test was conducted for the overall significance of the model and the value of p for the model was 0.014, indicating that the model was overall significant at the 5% level of significance.

**Table 4:** Analysis of variance.

Model		Sum of squares	df	Mean square	*F*	Sig.
1^a^	Regression	2.081	4	0.520	4.730	0.014^b^
	Residual	1.430	13	0.110		
	Total	3.510	17			

^a^Dependent variable: lnU. ^b^Predicted variable: (Constant), lnP, lnG, lnR, lnS.

According to the t-test for the significance of the regression parameters shown in Table 3, the value of p of government subsidies equals 0.001, indicating that government subsidies have a significant effect on the operating income of strategic emerging enterprises and eventually enter the regression equation, while the *p*-values of the other variables are all greater than 0.1 and do not pass the significance test, indicating that they have no significant effect on the dependent variable and do not enter the regression equation.

From the above analysis, the regression equation was derived as


(6) InU=0.63lnG+8.775+μ

Government subsidies have an important driving role in the development of strategic new industries. The result is closely related to the characteristics of strategic emerging industries. Strategic emerging industries have high input costs and slow returns. Without government support, it is difficult for enterprises to persist on their own.

In recent years, key areas such as new generation information technology, biology, high-end equipment, new materials, energy conservation and environmental protection, new energy, new energy vehicles and digital creativity have all achieved rapid development, driven by many new dynamic industries. However, behind the huge development prospect of strategic emerging industries is a huge amount of investment, the gathering of wisdom and the determination of business leaders. In the context of increasingly fierce international development competition and China’s development momentum shift, the development of strategic emerging industries faces more challenges, such as breakthroughs in key technologies, the rapid transformation of innovation results and cultivation and development of innovation-led emerging industries. Through the path analysis, we can find that the leading role of government subsidies is very prominent, the policy nature of strategic emerging industries is obvious, and the community pays more attention to the government’s “attitude” toward the development of strategic emerging industries.

## 6. Conclusion and discussion

### 6.1. Conclusion

The main objective of this study was to examine the regional differences in the efficiency of the integration of technology and finance in China. All regions of China have both provinces with high and low integration efficiency. Provinces in the relatively developed eastern and southern regions, while some provinces in the central region can also be at a high level of integration efficiency. The regression analysis reveals that government support is the most significant influence on new strategic industries, and government support is not only reflected in financial subsidies, but also in the optimisation of the business environment and credit guarantees for technology enterprises.

This study focuses on the three-stage efficiency measurement of 206 technology boards, and conducts a differential analysis of the efficiency of the integration of technology and finance in China. Unlike the previous cause-effect relationship or promotion-inhibition relationship deduced from the correlation factors, we mainly explore the differences in the development of technology and finance through the regions, and precisely and comprehensively understand the integration of technology and finance in each region of China, while the development of finance and technology is extremely crucial to the national economy. The development of finance and technology is extremely crucial to the development of the national economy (Zagorchev et al., 2011; Zhang and Chen, 2015).

### 6.2. High demand for financial support for the development of strategic emerging industries

Strategic emerging industries include basic research, platform construction, public services and other levels, and involve key areas such as new generation information technology, biology, high-end equipment, new materials, energy conservation and environmental protection, new energy, new energy vehicles and digital creativity, etc. They are typical areas where capital and technology gather and are in fierce competition with international industrial development, and their demand for financial support at all stages of their life cycle is huge and long-term. The demand for financial support at all stages of the life cycle is huge and long-term.

### 6.3. Regional differences in the development of strategic emerging industries are more obvious

The strength and prospect of the development of strategic emerging industries in the eastern coastal, southern coastal and central Yangtze River regions are all in the middle and high level of the country, with some regions having scale and some regions having efficiency, indicating that complementary patterns can be formed between regions and that a ladder development system of emerging industry clusters can be constructed in response to regional differences to create a clear division of labor and a mutually articulated pattern of industry cluster development. For the development of strategic emerging industries along the coast, accelerate the degree of opening up of emerging fields to the outside world, and strengthen the selection and training of innovative talents in the field of strategic emerging industries. Strengthen international innovation cooperation, actively integrate into the global innovation system, explore the sharing of innovation achievements and break down international market barriers to the application of new technologies.

### 6.4. Strong government guidance for the development of strategic new industries

Data from the National Bureau of Statistics shows that China’s economic development new kinetic energy index will grow at an average annual rate of 29.8% from 2015 to 2020. In the future, the digital economy, artificial intelligence, integrated circuits, big data, new energy vehicles, new energy equipment and power generation, biomedicine, intelligent robots and other emerging dynamic industries will all maintain rapid growth. The relationship between the government and the market is constantly adjusting, and the future development of strategic emerging industries requires a new division of labor between the government and the market. Government guidance is not only in the form of government subsidies, such as tax breaks and product purchase subsidies for new energy industries, but also in the transfer of the Internet economy to strategic emerging industries and the development of a variety of “Internet plus” innovative technologies. The “Key Technology Project Reveal System” was launched to accelerate the transformation of scientific and technological achievements. Research and transformation projects on socially beneficial and industry-general technologies should be given priority support to promote the transformation of core technologies and achievements urgently needed for industrial development.

### 6.5. Discussion

On the whole, the integration of technology and finance can effectively promote the improvement of production efficiency of micro enterprises. Therefore, relevant departments should further create policy space for the development of local strategic emerging industries, combine existing policy experience, increase the combination of technology and finance, innovate technology financial support mode, improve the quality of financial services, and further solve the financing constraints faced by the development of strategic emerging industries (Bash et al., 2014).

The current pilot policy of combining technology with finance has a more obvious effect on promoting the productivity of strategic emerging industries. Therefore, the transmission of technology finance policies needs to be combined with the specific development stages of strategic emerging industries, and through channels such as technology incubation parks and industrial clusters, the coverage of technology finance policy information should be expanded as far as possible to increase the participation of strategic emerging industries in the policy, thereby strengthening the implementation effect of technology finance policies.

Research and Development (R&D) is the focal point for the effective integration of technology with finance, thereby enhancing the competitiveness of firms, and therefore policy arrangements for the integration of technology with finance need to focus on the role of corporate innovation in productivity development (Antonio and Ugo, 2016; Hu, 2018). For example, government departments can further optimize the relevant system construction, actively play a guiding function, and lead social capital to participate in the investment field of innovation projects in strategic emerging industries; insurance institutions should actively explore the claim system for enterprise innovation risks, provide diversified insurance services, and gradually improve the risk-sharing mechanism to support strategic emerging industries, thus relieving the problem of insufficient financing for enterprise innovation activities.

The healthy and sustainable development of strategic emerging industries cannot be achieved without the synergistic cooperation between the government and the market. Along with the rapid development of new technologies and new business models in strategic emerging industries, the originally clear relationship between the government and the market is being adjusted, and the future development of strategic emerging industries requires a new division of labor between the government and the market. The development of strategic emerging industries provides many technologies that improve people’s living standards and quality, and the relationship with the government becomes mutually supportive and win-win. Many public services that were originally provided by the government alone are increasingly being provided jointly by the government and the market. For example, with the development of smart cities, more and more companies are becoming smart city operators, taking on the function of providing public services such as traffic management for cities (Peter and Gerhard, 2018). Another example is that with the rapid development of e-commerce, government market supervision responsibilities such as combating counterfeit and shoddy products are increasingly being accomplished by e-commerce platform companies in conjunction with regulatory authorities. At the same time, many business decisions that were originally made by enterprises themselves are beginning to require government intervention. For example, data privacy provisions between Internet enterprises and users are no longer purely commercial, and the determination of their relevant content requires direct government intervention (Nir, 2016). Another example is that the pricing of innovative drugs is not the result of maximizing the company’s own revenue, but is often agreed upon by the government and the company with reference to clinical value. In order to better promote the development of the industry in the future, the government needs to strengthen innovation, define its own position and provide appropriate service system.

### 6.6. Limitations and future work

The development of strategic emerging industries involves the development of enterprises in many special fields, and the cognition of innovation activities and transformation of achievements should be more comprehensive. 206 enterprises on the Science and Technology Innovation Board are used as the sample in this paper, which includes all kinds of strategic emerging enterprises as far as possible, but with the changes in society, policies, economy and technology, some enterprises are facing various development paths, such as mergers and restructuring, diversification, etc., so the tracking of the sample and the aggregation of the sample are somewhat limited. However, with changes in society, policy, economy and technology, some enterprises are facing various development paths, such as mergers and restructuring, diversification, etc., thus limiting the trackability and aggregation of the sample. In the future, this paper can carry out more detailed sector-specific research to analyze the deeper relationship between the regional economy and the field of technology, and to analyze more thoroughly the role and efficiency of financial support in the whole process of technology development.

## Data availability statement

The original contributions presented in the study are included in the article/supplementary material, further inquiries can be directed to the corresponding author.

## Author contributions

QY: conceptualization, validation, resources. LC: methodology and software. XW: formal analysis, investigation, and writing – original draft preparation. QG: data curation. KZ: visualization. XL: writing – review and editing. All authors have read and agreed to the published version of the manuscript.

## Conflict of interest

The authors declare that the research was conducted in the absence of any commercial or financial relationships that could be construed as a potential conflict of interest.

## Publisher’s note

All claims expressed in this article are solely those of the authors and do not necessarily represent those of their affiliated organizations, or those of the publisher, the editors and the reviewers. Any product that may be evaluated in this article, or claim that may be made by its manufacturer, is not guaranteed or endorsed by the publisher.
